# Influence of Raw‐Material Processing on Gold and Silver Nanoparticles Stability Using Ultrasound‐Extracted Pink‐Pepper Compounds

**DOI:** 10.1002/cbdv.202501093

**Published:** 2025-08-22

**Authors:** Lucas Cantalovo Pereira, Gabriel César Resende, Anderson de J. Gomes, Claure Nain Lunardi

**Affiliations:** ^1^ Laboratory of Photochemistry and Nanobiotechnology University of Brasília Brasília Brazil

**Keywords:** gold nanoparticles, green synthesis, *Schinus terebinthifolius*, silver nanoparticles, ultrasound‐assisted extraction

## Abstract

The ultrasound‐assisted green synthesis of gold (AuNPs) and silver nanoparticles (AgNPs) from *Schinus terebinthifolius* extracts delivered stable colloids whose physicochemical properties depend on raw‐material preprocessing. Ground sonicated extracts (GSE) produced narrower size distributions and more negative zeta potentials (−19.6 mV for AuNPs, −23.5 mV for AgNPs) than unprocessed sonicated extracts (USE), which favored aggregation, especially at 100°C. Surface‐plasmon resonance (SPR) red‐shifted for AuNPs‐USE, indicating larger particles. High‐resolution transmission electron microscopy (TEM) corroborated these trends: GSE‐derived AgNPs formed 15–40 nm quasi‐spheres, whereas USE yielded fused dendritic assemblies of 20–80 nm subunits; AuNPs ranged from densely coalesced 8–25 nm chains (100°C) to isolated spheres (25°C). Coherent lattice fringes with *d* = 0.23 nm {1 1 1} and 0.20 nm {2 0 0} confirmed phase‐pure fcc structures, and fringe continuity across AgNP necks evidenced oriented attachment. Collectively, the data show that extract grinding enhances nucleation control, crystallinity, and colloidal stability, providing a scalable route to biogenic AuNPs and AgNPs for biomedical, catalytic, and food‐preservation applications.

## Graphical abstract

*Schinus terebinthifolius* extract and metal nanoparticle synthesis.

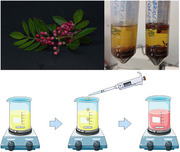

## 1 Introduction

This study is the first to systematically compare ground and unprocessed pink‐pepper berries as renewable feedstocks for green Au and Ag nanoparticle synthesis, revealing that milling increases surface area, accelerates phytochemical release, and yields smaller, less aggregated colloids, whereas intact berries produce larger, more stable particles; by showing how raw‐material processing can replace hazardous reagents with low‐toxicity biomass, boost yields, reduce waste, and energy consumption, harness intrinsic plant extracts as natural reductants/catalysts, and valorize agricultural by‐products within a circular‐economy framework, the work establishes feedstock form as a decisive lever for sustainable nanomaterial production.

Nanotechnology has revolutionized various scientific fields, including biomedicine, catalysis, and environmental science [1, 2]. Among different nanomaterials, gold (AuNPs) and silver nanoparticles (AgNPs) have garnered significant attention due to their unique physicochemical properties, such as high surface‐area‐to‐volume ratios, tunable optical characteristics, and excellent biocompatibility [3].

However, conventional synthesis methods often involve hazardous chemicals and energy‐intensive processes, necessitating the development of environmentally sustainable approaches [4]. Green synthesis, which leverages plant‐derived bioactive compounds as reducing and stabilizing agents, offers an eco‐friendly alternative with enhanced biocompatibility and reduced environmental impact [5].

Green synthesis has emerged as a sustainable and eco‐friendly alternative to conventional nanoparticle fabrication methods, offering significant advantages in terms of environmental safety, cost‐effectiveness, and biocompatibility. Unlike traditional physical and chemical approaches that often involve toxic reagents and high energy consumption, green synthesis utilizes biological resources such as plant extracts as reducing and stabilizing agents, enabling the formation of metal and metal oxide nanoparticles under mild conditions. For instance, Durmaz et al. [6] demonstrated the successful synthesis of ZnO nanoparticles using *Euphorbia stricta* L. extract via a supercritical CO‐assisted microwave hydrothermal method, highlighting their potent antibacterial and photocatalytic properties against organic dyes and pathogens. Similarly, Hussaini et al. [7] employed different parts of the *Lupinus pilosus* plant to produce ZnO micro/nanorods, which exhibited promising optoelectronic performance in UV photodetector applications. Expanding on this, Ulukuş et al. [8] synthesized selenium‐doped ZnO and Ag/AgO nanocomposites using *Pinus nigra* pollen extract, achieving enhanced photocatalytic degradation of tetracycline and methylene blue, along with notable antibacterial activity. These studies collectively underscore the versatility and effectiveness of green synthesis in producing functional nanomaterials for environmental and biomedical applications.

**FIGURE 1:**
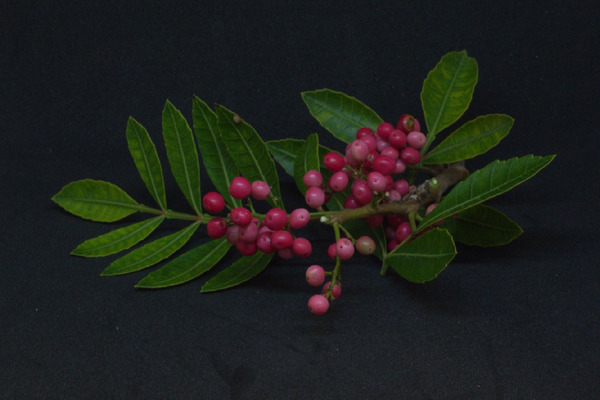
Photo of *Schinus terebinthifolius* (pink pepper), highlighting the leaves, branches, and characteristic reddish berries of this species from the Anacardiaceae family.

*Schinus terebinthifolius* (pink pepper) is widely recognized for its rich phytochemical profile, including essential oils, flavonoids, and phenolic compounds, which exhibit potent antioxidant and antimicrobial properties [9]. These bioactive compounds make *S. terebinthifolius* a promising candidate for the green synthesis of metal nanoparticles. Although prior research has explored plant‐mediated nanoparticle synthesis, the impact of raw‐material processing on nanoparticle stability and physicochemical characteristics remains underexplored. The image of *S. terebinthifolius* below (Figure 1) highlights the plant's characteristic leaves, branches, and reddish berries, which are the primary sources of bioactive compounds utilized in this study.

**FIGURE 2:**
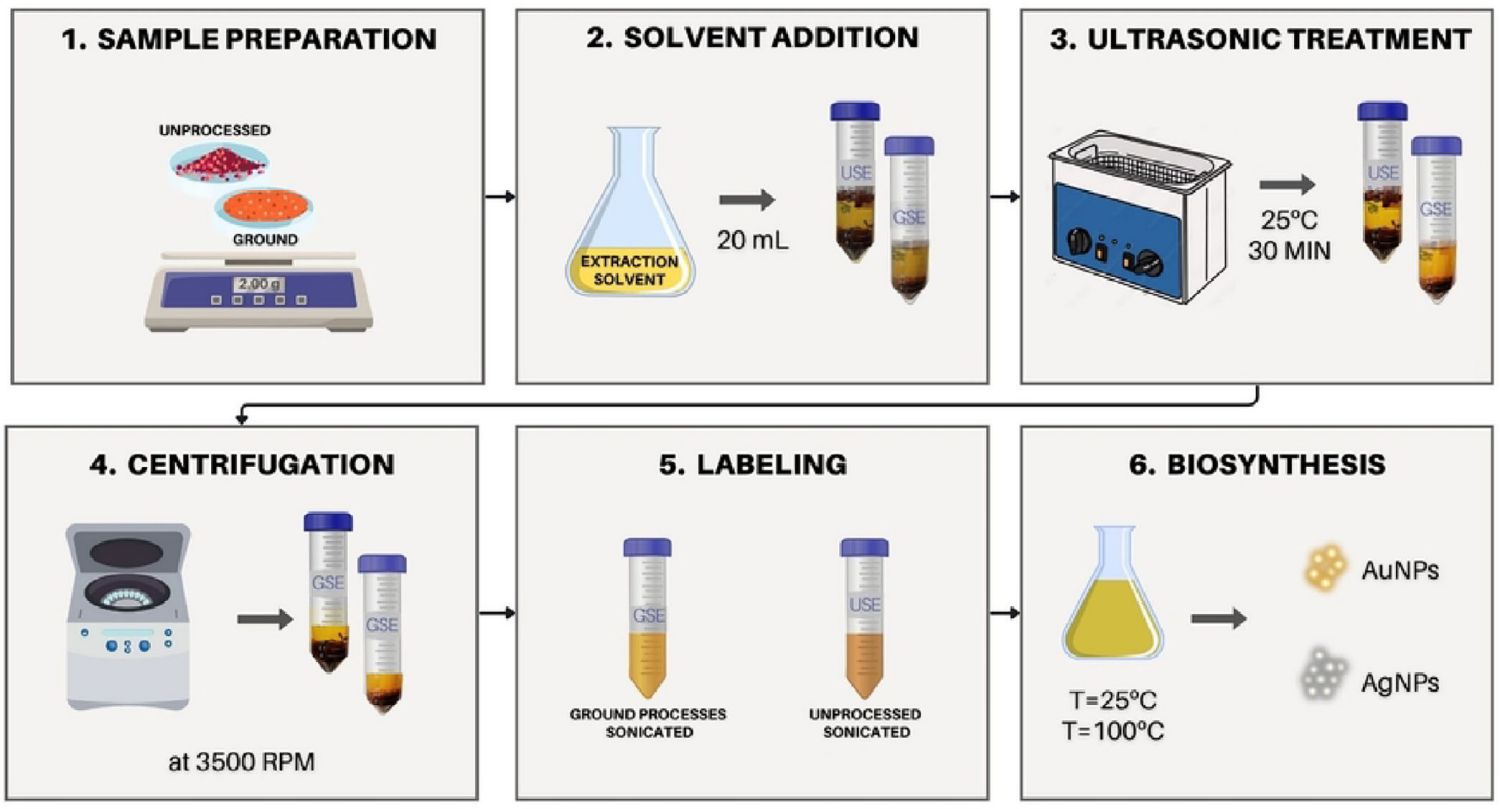
Schematic representation of the extraction procedure.

**FIGURE 3:**
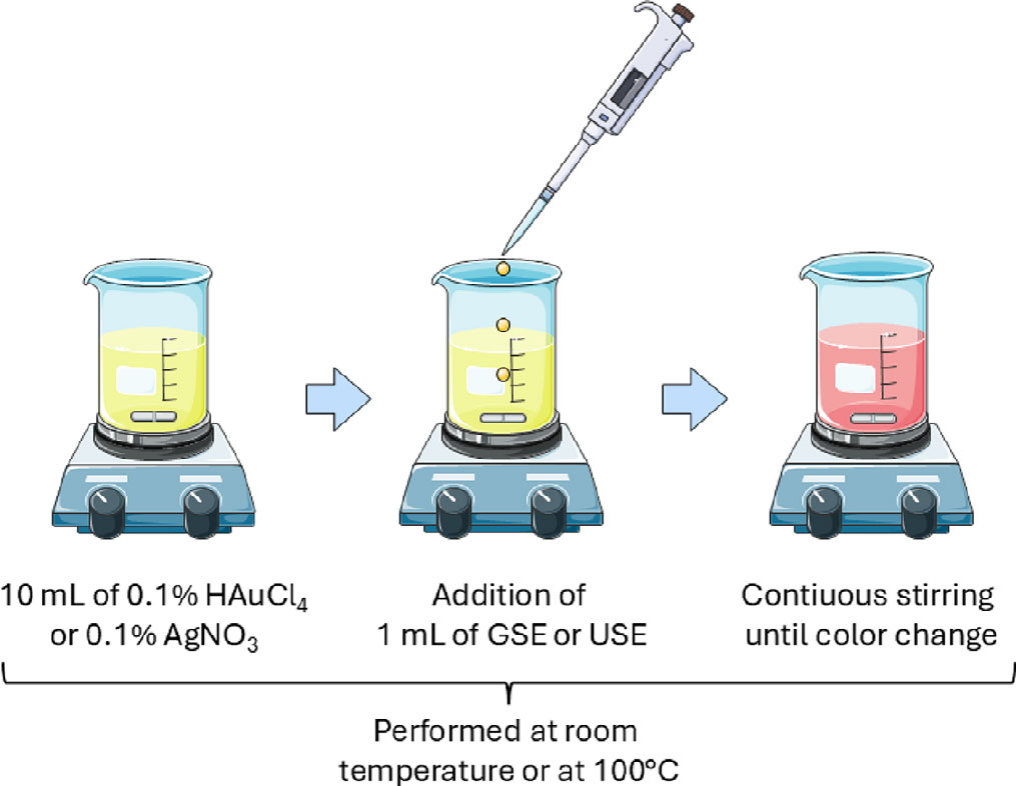
Schematic representation of the biosynthesis of metallic nanoparticles from pink‐pepper extract. GSE, ground‐sonicated extracts; USE, unprocessed sonicated extracts. *Source*: Image adapted from Servier Medical Art (https://smart.servier.com/), licensed under CC BY 4.0 (https://creativecommons.org/licenses/by/4.0/).

### 1.1 Ultrasound‐Assisted Extraction (UAE): A Sustainable Approach

To efficiently extract bioactive compounds from *S. terebinthifolius*, UAE was employed. UAE has emerged as an efficient and sustainable alternative to conventional extraction techniques, such as steam distillation and maceration. This method enhances extraction yield by facilitating cell wall disruption, improving mass transfer, and significantly reducing extraction time. Additionally, UAE operates at lower temperatures, preserving thermosensitive compounds, such as flavonoids and essential oils, which maintain their antioxidant and antimicrobial properties [9]. Moreover, the method requires minimal solvent use, aligning with green chemistry principles and reinforcing its suitability for eco‐friendly nanoparticle synthesis.

Natural materials present complex and intricate structures and contain many different active components in each of its parts, as a result the processing state of the material may impact directly the chemical profile of the resulting extraction and consequently the physicochemical characteristics of any nanoparticles obtained with it. Therefore, this study compares the synthesis and stability of gold and silver nanoparticles prepared utilizing different extracts of *S. terebinthifolius* and presence of heating in order to investigate the influence of raw‐material processing in the formation and characteristics of the nanoparticles. By elucidating the impact of raw‐material states before extraction, on nanoparticle formation and aggregation tendencies, this research contributes to optimizing green nanotechnology applications in biomedicine, catalysis, and food‐preservation.

## 2 Materials and Methods

### 2.1 Materials

To ensure consistency in phytochemical composition, *S. terebinthifolius* (pink pepper) dry berries were sourced from the commercial supplier Zona Cerealista (São Paulo) in January of 2024, and the lot number was 1670. All reagents, including hydrogen tetrachloroaurate (HAuCl_4_) and silver nitrate (AgNO_3_), were of analytical grade and purchased from Sigma‐Aldrich. Deionized water was used for all experimental procedures to prevent contamination. To access traditional knowledge: National System for the Management of Genetic Heritage and Associated Traditional Knowledge (SisGen), registration number A301E7A.

### 2.2 Essential Oils Extract via UAE

UAE was employed to obtain bioactive compounds from *S. terebinthifolius* essential oil extract. Two different sample preparations were tested to evaluate the influence of raw‐material processing:

Ground sample (G): Dry pink‐pepper berries were manually ground using a mortar and pestle until every berry had its pulp fully separated from its core.

Unprocessed sample (U): Whole dry pink‐pepper berries were used without any pretreatment.

### 2.3 Extraction Procedure

For each sample type, the following extraction procedure was conducted:

Sample preparation: An amount of 2.00 g of either ground or unprocessed dry pink‐pepper berries was placed in a 50‐mL centrifuge tube.

Solvent addition: A volume of 20 mL of extraction solvent (80% methanol) was added to the tube.

Ultrasonic treatment: The samples were subjected to an ultrasonic bath (40 kHz) for 30 min at 25°C to enhance the release of bioactive compounds.

Centrifugation: The extracted solution was centrifuged at 3500 rpm for 5 min to separate particulates.

The extract solution was labeled according to this origin as GSE (ground‐sonicated extract) or USE (unprocessed sonicated extract). All extraction procedure were schematically displayed in Figure 2.

### 2.4 Biosynthesis of Gold and Silver Nanoparticles (AuNPs and AgNPs)

The synthesis of gold and silver nanoparticles was carried out using the UAE‐derived extracts as reducing and stabilizing agents; as such extracts are composed of many volatile substances, temperature might affect the chemical profile of the final product; therefore, in order to evaluate the impact of heating, two synthesis conditions were tested:

Room temperature (rt): Nanoparticle formation at ambient temperature.

Heated condition (hot): Nanoparticle formation under heating conditions (100°C).

For each synthesis condition, the following protocol was applied.

#### 2.4.1 Reaction Mixture Preparation

One mL of pink‐pepper extract from GSE or USE was mixed with 10 mL of either 0.1% HAuCl_4_ (for AuNP synthesis) or 0.1% AgNO_3_ (for AgNP synthesis). The reaction mixture was stirred continuously under the specified temperature conditions. The formation of nanoparticles was confirmed visually by color changes (purple/red for AuNPs and yellow/brown for AgNPs) displayed in Figure 3.

### 2.5 Characterization of Nanoparticles

The physicochemical properties of the synthesized nanoparticles were assessed using multiple analytical techniques. UV–Vis spectroscopy was utilized to identify the surface‐plasmon resonance (SPR) of AuNPs and AgNPs, which was measured to be in the range of 300–700 nm [3]. DLS was used to verify particle size distribution and polydispersity index (PdI) in order to evaluate nanoparticle uniformity. Zeta potential was measured to determine the electrostatic stability of the nanoparticles. Fourier‐transform infrared spectroscopy (FTIR) was analyzed to confirm functional group involvement in nanoparticle stabilization, and it also displayed the interaction between plant‐based bioactive compounds and nanoparticle surfaces. For high‐resolution transmission electron microscopy (HRTEM), suspensions (5 µL) were drop‐cast onto carbon‐coated copper grids (300 mesh) and air‐dried. HRTEM images were acquired on a Tecnai G2 20 (FEI) transmission microscope operating at 200 kV. Lattice spacings were measured from the HRTEM micrographs by fast Fourier transform analysis using ImageJ.

### 2.6 Statistical Analysis

All experiments were conducted in triplicate to ensure reproducibility. Results are presented as mean ± standard deviation. Statistical significance was determined using one‐way analysis of variance (ANOVA), with a significance level set at *p* < 0.05.

## 3 Results and Discussion

### 3.1 Influence of Raw‐Material Processing and Temperature on Nanoparticle Properties

**FIGURE 4:**
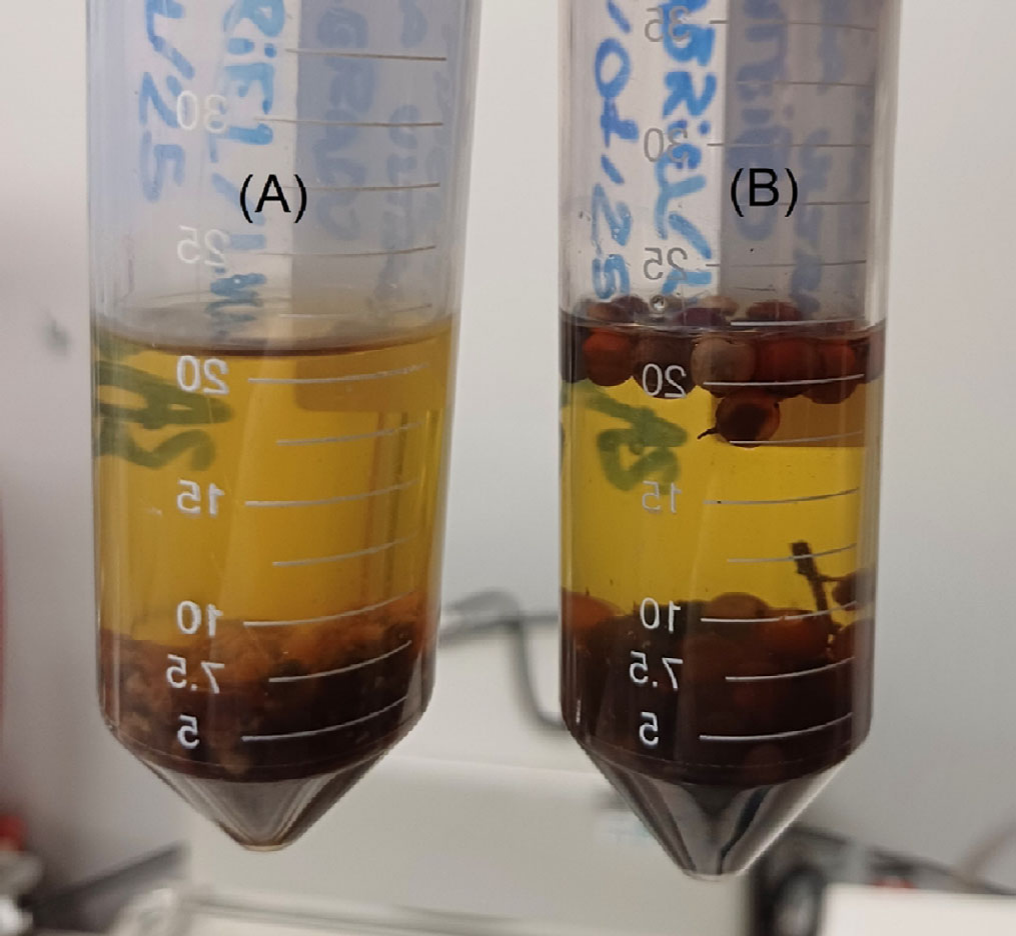
(A) GSE and berries and (B) USE and berries.

On the basis of an extensive literature review, UAE was identified as the optimal method for obtaining bioactive compounds. Additionally, the raw material was classified into two distinct forms for further investigation: GSE and USE samples. Figure 4 illustrates the extract from both samples.

**FIGURE 5:**
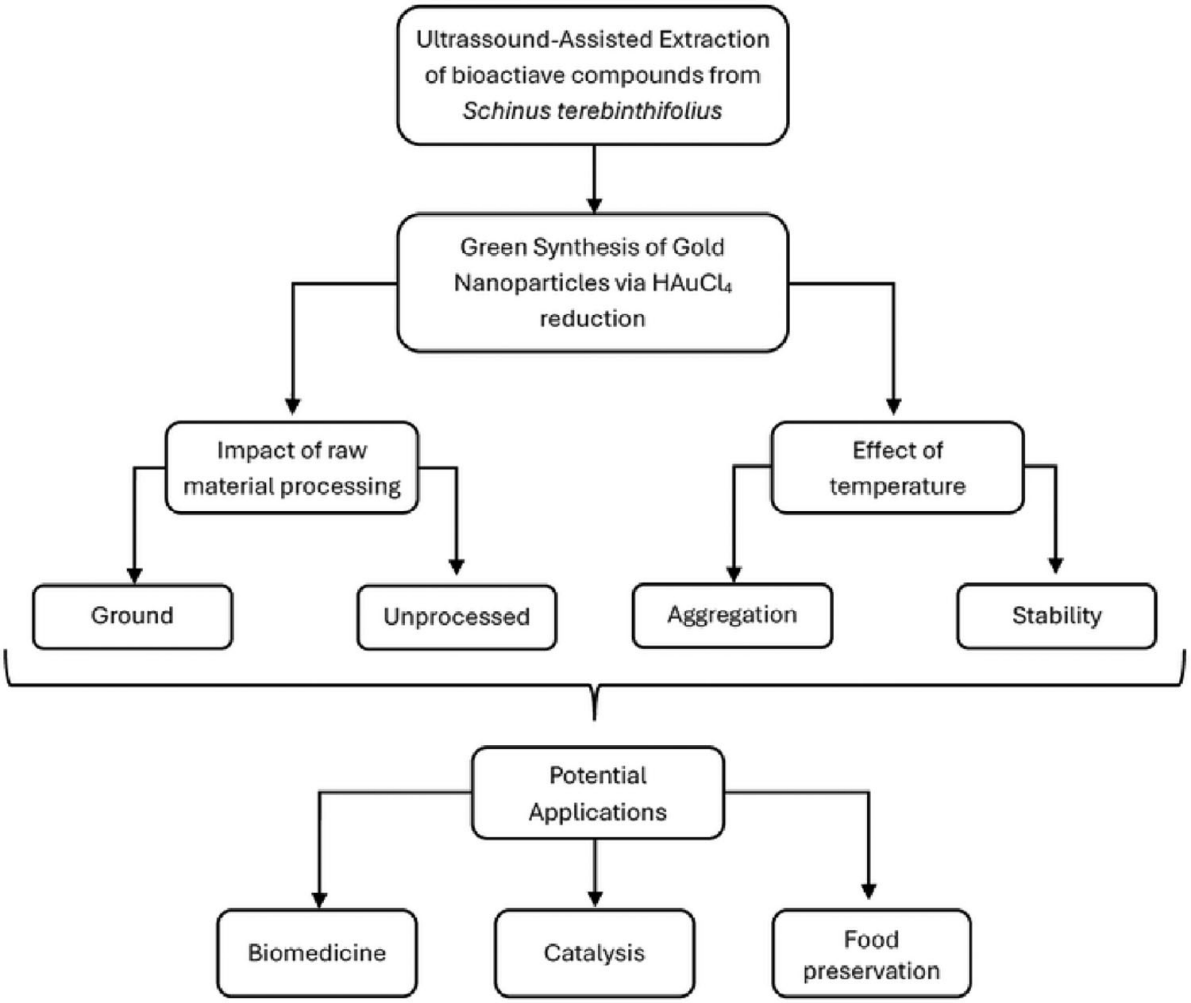
Flowchart for the evaluation of the biosynthesis of metallic nanoparticle from pink‐pepper extract.

The synthesis of gold or silver nanoparticles (AuNP/AgNP) was performed using UAE from *S. terebinthifolius* berries to compare the effects of GS and US. The impact of temperature variation (rt vs. hot) on nanoparticle stability and characteristics was also evaluated. Prior research has established the effectiveness of phytochemicals (e.g., flavonoids and phenolics) in NP stabilization [3]. However, few studies have examined how raw‐material processing (G vs. U) influences NP formation and stability. This study fills this knowledge gap, demonstrating that preprocessing enhances colloidal stability by modulating particle size, charge distribution, and aggregation behavior [10, 11] as represented in flowchart (Figure 5).

### 3.2 Nanoparticle Formation and Optical Properties (SPR Analysis)

**FIGURE 6:**
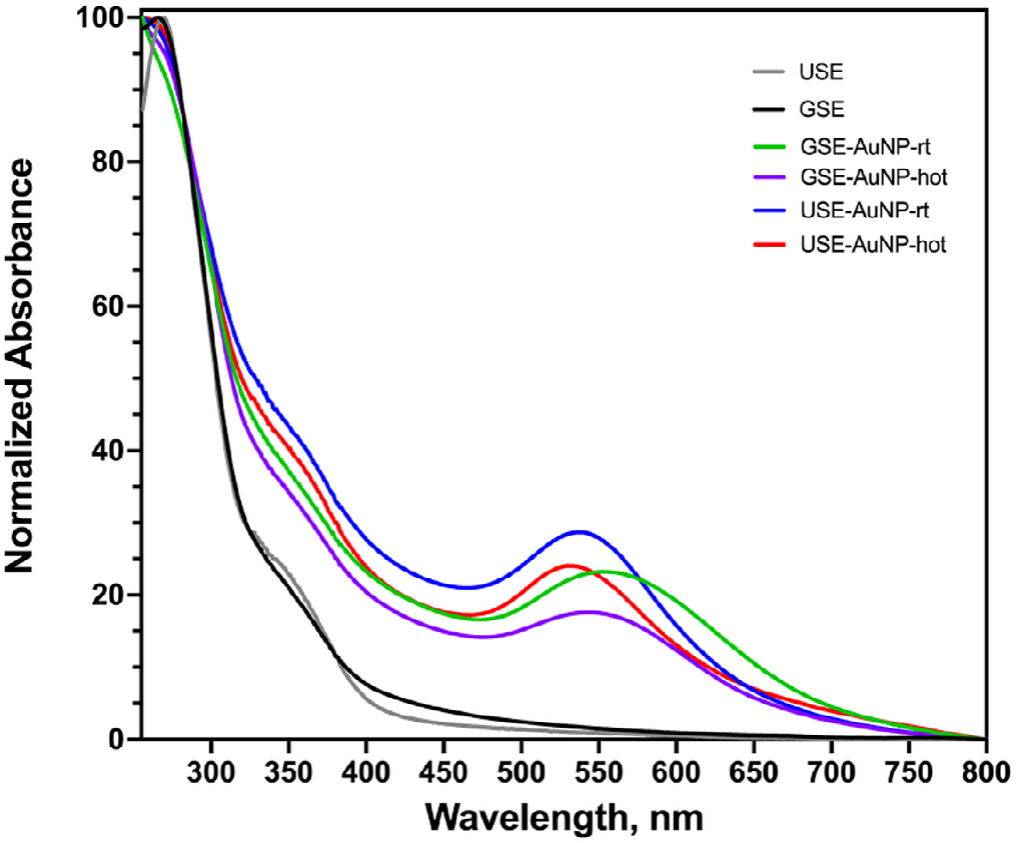
Normalized UV–Vis spectra of biosynthesis AuNP from USE and GSE. GSE, ground‐sonicated extracts; USE, unprocessed sonicated extracts.

The formation of AuNPs was confirmed by UV–Vis spectroscopy, where SPR peaks were observed between 530 and 600 nm. A distinct redshift was noted in nanoparticles synthesized from USE, indicating larger particle size or increased aggregation. In contrast, GSE‐derived AuNPs exhibited a sharper SPR peak (∼530 nm), suggesting smaller, more monodisperse particles (Figure 6).

**FIGURE 7:**
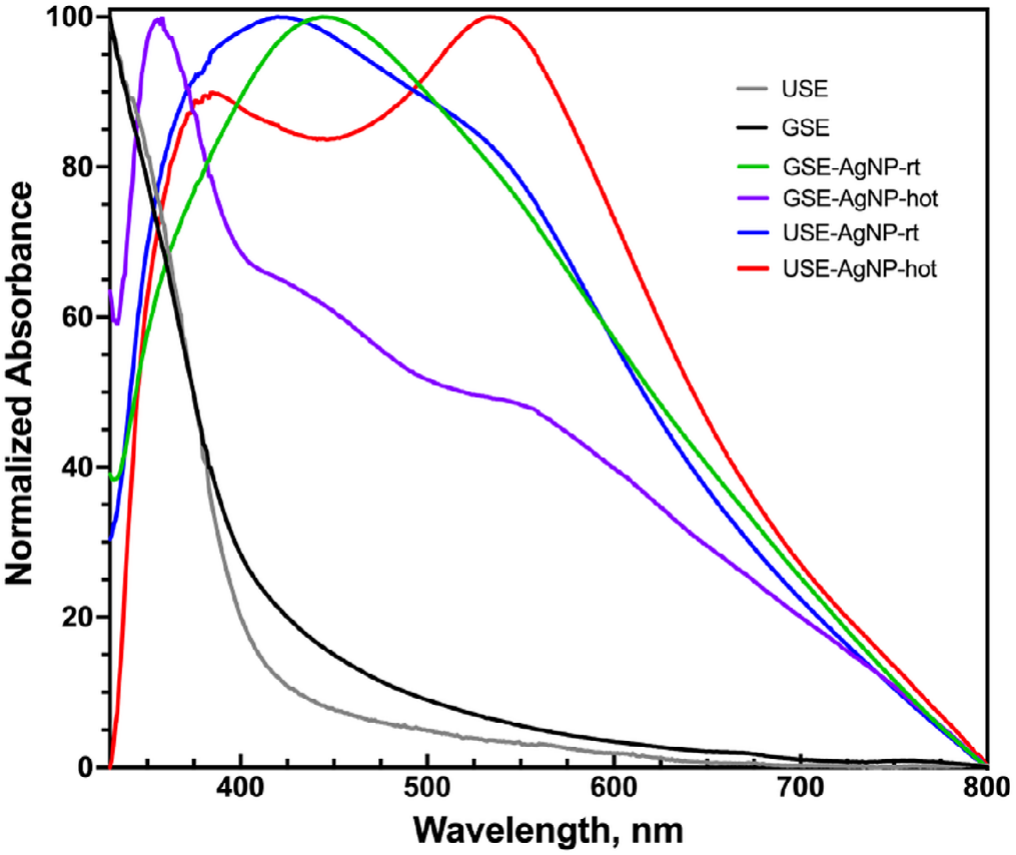
Normalized UV–Vis spectra of biosynthesis AgNP from USE and GSE. GSE, ground‐sonicated extracts; USE, unprocessed sonicated extracts.

SPR analysis of silver nanoparticles displayed characteristic peaks around 400–450 nm, depending on the particle size and aggregation state. The peak position shifted slightly toward longer wavelengths for larger or more aggregated particles. The sharper peaks observed for GSE‐derived AgNPs suggest more uniform particle formation, whereas broader peaks for USE‐derived AgNPs indicate increased size variability and aggregation tendencies (Figure 7).

### 3.3 Size, Zeta, and PdI of All Nanoparticles

#### 3.3.1 Gold Nanoparticles

The particle size of AuNPs was analyzed to assess their uniformity and stability under different synthesis conditions. The results indicate that GSE‐AuNP‐hot (116 nm) exhibited the smallest average size, suggesting a more controlled and stable nanoparticle formation. In contrast, USE‐AuNP‐hot (143 nm) displayed a larger particle size, indicating a greater tendency for aggregation. The increase in size in USE‐AuNP‐hot may be attributed to the synthesis conditions, leading to reduced colloidal stability and increased nanoparticle clustering. Zeta potential measurements were performed to evaluate the electrostatic stability of the synthesized nanoparticles [12]. More negative zeta potential values indicate stronger electrostatic repulsion, reducing the likelihood of aggregation. The GSE‐AuNP‐hot sample exhibited a zeta potential of −19.6 mV, suggesting superior colloidal stability. Conversely, the USE‐AuNP‐hot sample presented a significantly lower zeta potential of −7.2 mV, which indicates weaker repulsive forces and a higher probability of aggregation. The PdI was measured to determine the uniformity of nanoparticle distribution within the colloidal solution. A lower PdI value indicates a more monodisperse and stable nanoparticle population. The GSE‐AuNP‐hot sample demonstrated the best uniformity with a PdI of 0.165, signifying a highly homogeneous dispersion. In contrast, USE‐AuNP‐hot exhibited a significantly higher PdI of 0.450, indicating a broad size distribution and greater aggregation tendencies. This suggests that the synthesis parameters for USE‐AuNP‐hot resulted in less controlled nucleation and growth, contributing to higher polydispersity.

**FIGURE 8:**
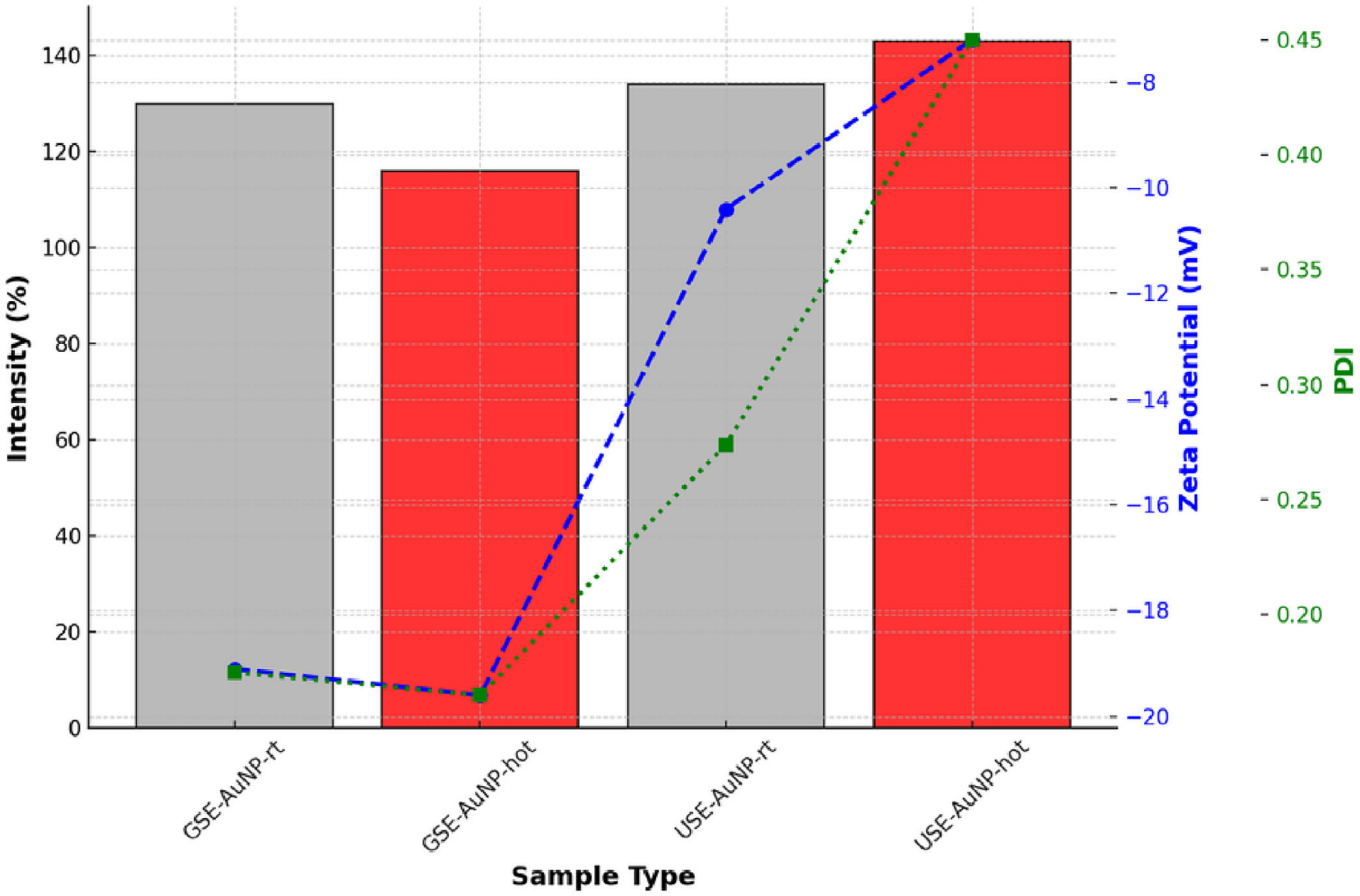
Size, zeta, and PdI of biosynthesis AuNP from USE and GSE. GSE, ground‐sonicated extracts; PdI, polydispersity index; USE, unprocessed sonicated extracts.

GSE‐AuNP‐hot demonstrated superior stability, smaller particle size, and better uniformity, making it a more suitable candidate for applications requiring stable and monodisperse nanoparticles. In contrast, USE‐AuNP‐hot exhibited a larger particle size, lower zeta potential, and higher polydispersity, indicating a greater tendency toward aggregation (Figure 8).

#### 3.3.2 Silver Nanoparticles

The particle size of AgNPs was evaluated to determine their distribution and aggregation tendencies. The results indicate that GSE‐AgNP‐hot exhibited the largest particle size at 731 nm, suggesting increased particle growth due to synthesis conditions. In contrast, USE‐AgNP‐rt was the smallest at 163 nm, implying more controlled nucleation and limited aggregation in room temperature conditions. Zeta potential measurements of AgNPs gave higher negative values corresponding to better colloidal stability due to stronger electrostatic repulsion. GSE‐AgNP‐hot demonstrated the highest stability with a zeta potential of −23.5 mV, ensuring a well‐dispersed colloidal solution. On the other hand, USE‐AgNP‐hot exhibited a less negative zeta potential of −19.6 mV, indicating a reduced electrostatic repulsion and a higher likelihood of aggregation.

**FIGURE 9:**
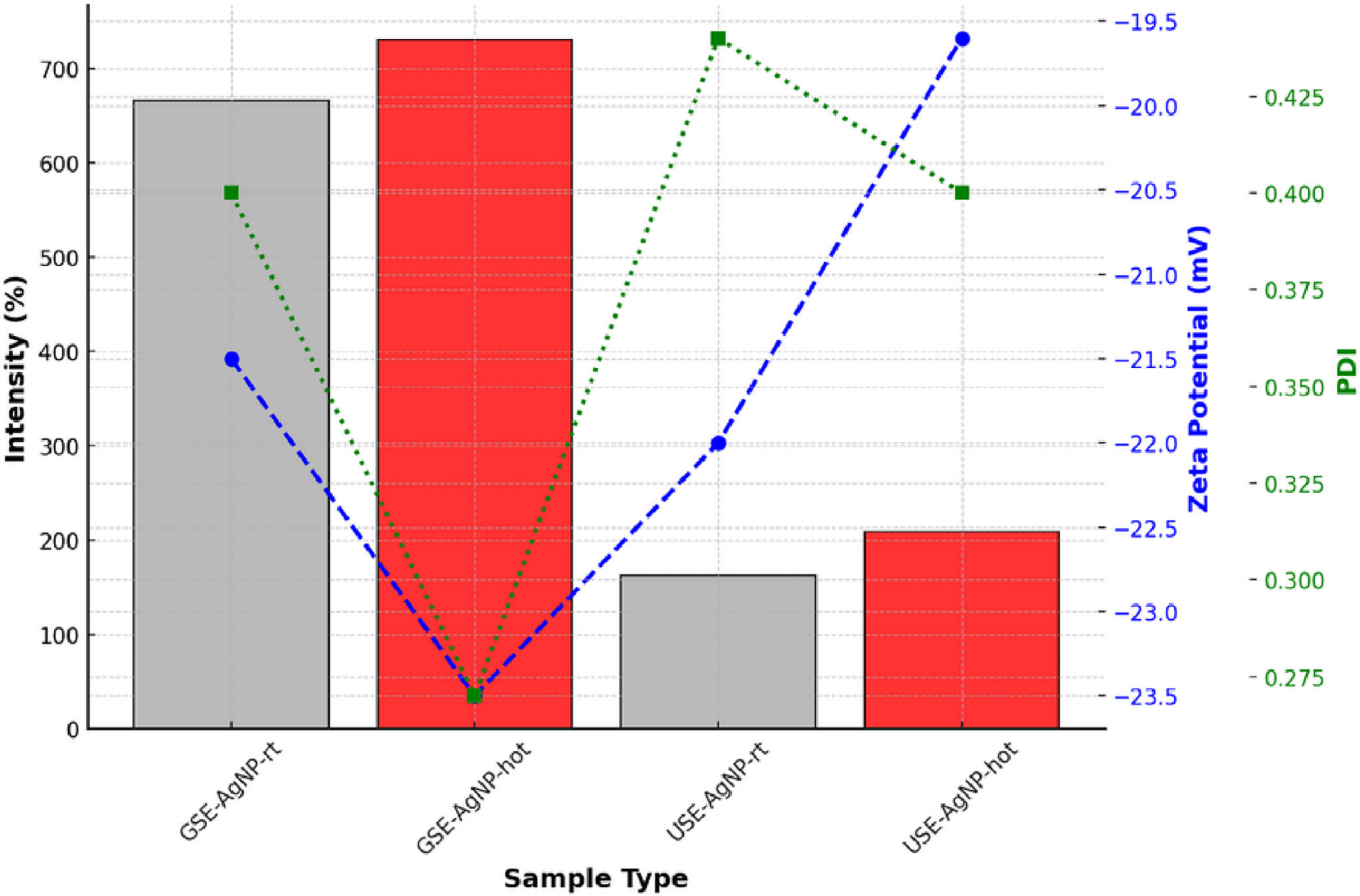
Size, zeta, and PdI of biosynthesis AgNP from USE and GSE. GSE, ground‐sonicated extracts; PdI, polydispersity index; USE, unprocessed sonicated extracts.

GSE‐AgNP‐hot had the best uniformity with a PdI of 0.27, indicating a well‐controlled synthesis process. In contrast, USE‐AgNP‐rt presented a higher PdI of 0.44, signifying a broader size distribution and increased aggregation tendencies (Figure 9).

The comparison between both major characterization results of AgNP highlights significant differences in particle size, stability, and uniformity based on synthesis conditions. GSE‐AuNP‐hot emerged as the most stable and uniform sample, with a larger particle size and superior zeta potential, making it suitable for applications requiring long‐term colloidal stability. In contrast, USE‐AgNP‐rt was the smallest but exhibited higher polydispersity, making it more prone to aggregation. These findings underscore the importance of optimizing synthesis parameters to tailor nanoparticle properties for specific applications (Table 1).

**TABLE 1:** summarizes all data from size, zeta, and polydispersity index (PdI).

Sample	Type	Size (nm)	Zeta potential (mV)	PdI	Behavior
GSE‐AuNP‐rt	Gold	130	−19.1	0.175	Small, stable, uniform
GSE‐AuNP‐hot	Gold	116	−19.6	0.165	Most stable AuNP, lowest PdI
USE‐AuNP‐rt	Gold	134	−10.4	0.274	Moderate stability, some aggregation
USE‐AuNP‐hot	Gold	143	−7.2	0.450	Highly polydisperse, worst stability
GSE‐AgNP‐rt	Silver	666.7	−21.5	0.400	Very large, moderately stable
GSE‐AgNP‐hot	Silver	731	−23.5	0.270	Most stable AgNP, larger but uniform
USE‐AgNP‐rt	Silver	163	−22.0	0.440	Smallest AgNP, high PdI (less uniform)
USE‐AgNP‐hot	Silver	209	−19.6	0.400	Slightly larger, reduced stability

Abbreviations: GSE, ground‐sonicated extracts; USE, unprocessed sonicated extracts.

These results indicate that mechanical disruption of plant material improves the extraction of bioactive compounds, enhancing NP stabilization. This aligns with findings by Suman et al. [4], who reported that plant extract composition affects NP synthesis efficiency.

### 3.4 FTIR Nanoparticle Analysis

**FIGURE 10:**
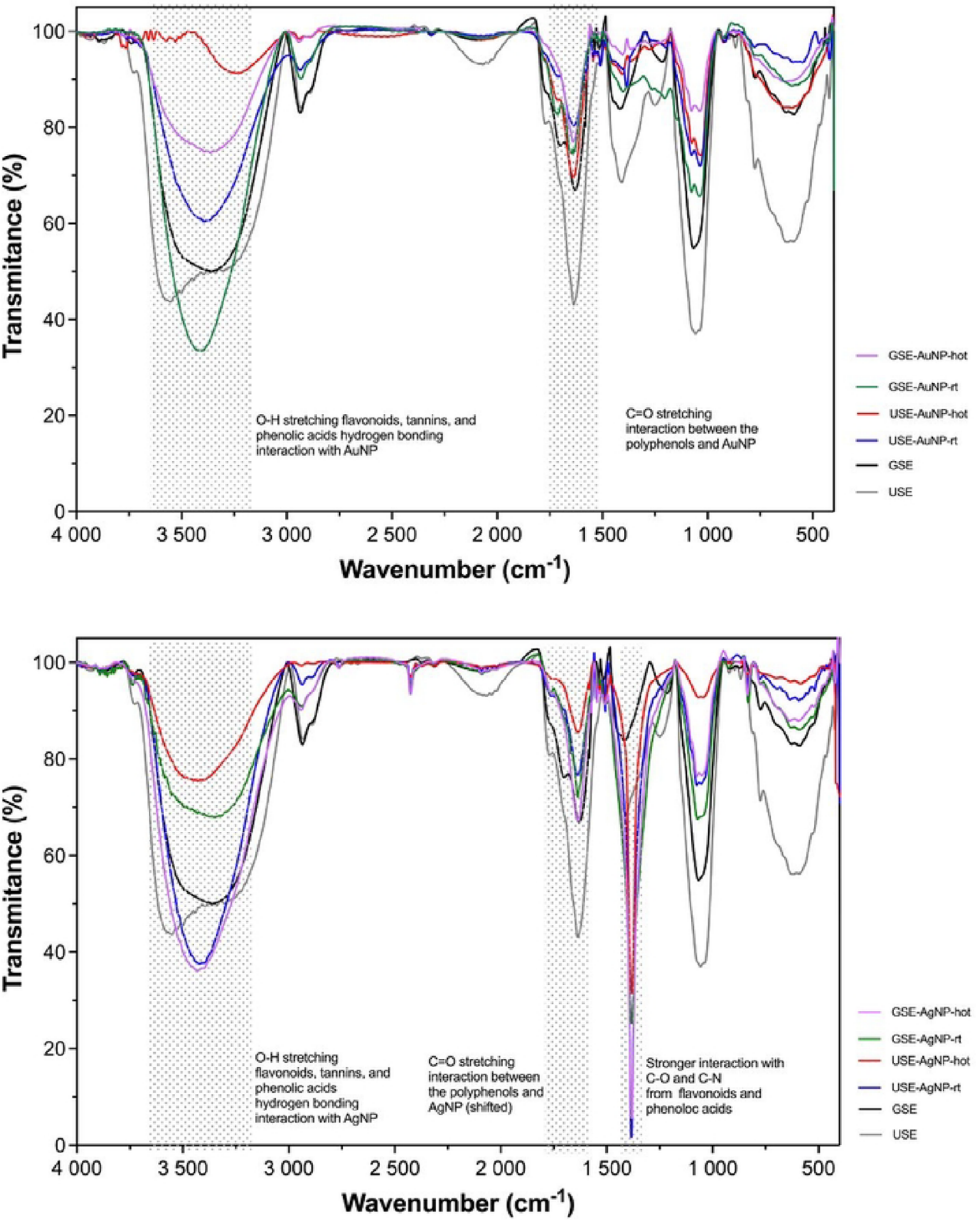
Fourier‐transform infrared spectroscopy (FTIR) of USE, GSE, and biosynthesis of AuNP and AgNP from USE and GSE. GSE, ground‐sonicated extracts; USE, unprocessed sonicated extracts.

FTIR spectroscopy revealed distinct binding characteristics, with AgNPs exhibiting stronger interactions with hydroxyl and carbonyl groups than AuNPs as shown in Figure 10A, B.

The FTIR spectrum of the gold sample exhibited strong absorption bands at 3322, 3312, and 3250 cm^−1^, corresponding to the O–H stretching vibrations of hydroxyl groups. These peaks are characteristic of flavonoids, tannins, and phenolic acids, which are key bioactive compounds present in pink‐pepper extract [13]. The presence of these peaks in the AuNP suggests hydrogen bonding interactions between the hydroxyl functional groups of polyphenols and the gold surface.

The C═O stretching vibrations were observed at 1713 and 1701 cm^−1^, indicative of carbonyl groups from ketones, aldehydes, esters, or carboxyl compounds. These functional groups are commonly found in gallic acid, caffeic acid, and flavonoids, which are known constituents of pink‐pepper extract. The shift in the carbonyl peak between the USE and the AuNP suggests a chemical interaction between them, likely through chelation or coordination bonding.

The FTIR spectrum of the AgNP sample revealed similar O–H stretching bands at 3322, 3312, and 3250 cm^−1^, confirming the presence of phenolic compounds from the pink‐pepper extract. However, the peak intensities and sharpness differed from the AuNP spectrum, suggesting stronger interactions between AgNP and hydroxyl groups, possibly due to enhanced hydrogen bonding or coordination effects.

Distinct C═O stretching vibrations were detected at 1713 and 1701 cm^−1^, similar to those observed in the gold spectrum. However, in the silver FTIR, these peaks were more intense and slightly shifted, indicating a stronger binding affinity of carbonyl groups with silver nanoparticles. This suggests that silver nanoparticles may exhibit preferential complexation with phenolic acids and flavonoids, possibly forming stronger metal‐ligand interactions compared to gold (Table 2).

**TABLE 2:** Comparison between the biosynthesis of AuNP and AgNP.

FTIR feature	AuNP	AgNP	Stability correlation
O–H stretch (∼3300 cm^−1^)	Present, broad peaks (moderate hydrogen bonding)	Present, sharper peaks (strong hydrogen bonding)	More defined peaks in AgNP suggest stronger interaction, which may enhance stability
C=O Stretch (∼1700 cm^−1^)	Moderate intensity, minor shifts	Stronger intensity, more defined shifts	AgNP shows stronger complexation, which may contribute to higher stability
Zeta potential (mV)	Closer to neutral (e.g., −10 to −20 mV)	Higher absolute values (e.g., −30 to −50 mV)	AgNP may exhibit better stability due to stronger surface charge repulsion
Binding strength	Moderate interaction with polyphenols	Stronger coordination with polyphenols	Stronger binding in AgNP could lead to better dispersion and reduced aggregation

A comparative analysis between AgNP and GSE suggests that AgNP exhibits stronger and more defined peaks in the hydroxyl and carbonyl regions, supporting the hypothesis that AgNP has a higher affinity for polyphenolic compounds than AuNP. The observed peak shifts and intensities confirm selective adsorption and complexation, which could enhance the stability and bioactivity of silver surface‐polyphenol nanocomposites.

A direct comparison between the AuNP and AgNP revealed key differences in peak intensity, sharpness, and shifts, indicating distinct interactions between pink‐pepper polyphenols and the metal surfaces:

These results indicate that both AuNP and AgNP interact with bioactive compounds in pink‐pepper extract, but AgNP exhibits a stronger and more defined interaction with hydroxyl and carbonyl groups. This difference may be attributed to the higher affinity of AgNP for oxygen‐containing functional groups, which can influence the stability and potential biological activity of the resulting nanocomposites. The study confirms that optimizing raw‐material preparation is crucial in plant‐mediated NP synthesis.

### 3.5 HRTEM Nanoparticle Analysis

**FIGURE 11:**
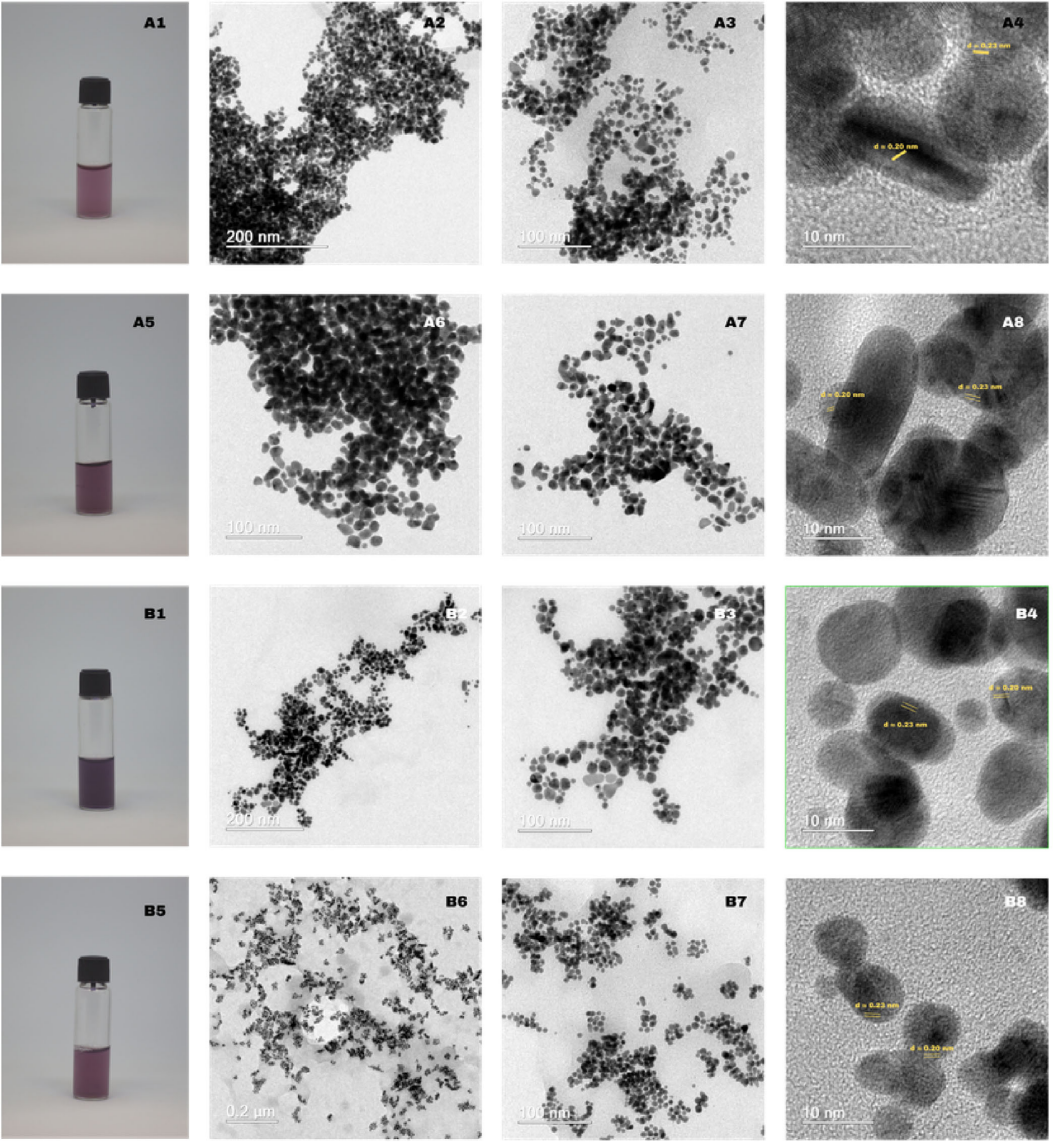
Colloidal solution image and HRTEM images for USE‐AuNP‐rt (A1—colloidal solution; A2–A4 (scale bar: 200, 100, 10 nm)) and USE‐AuNP‐hot (A5—colloidal solution; A6–A8 (scale bar: 200, 100, 10 nm)). GSE‐AuNP‐rt (B1—colloidal solution; B2–B4 (scale bar: 200, 100, 10 nm)) and GSE‐AuNP‐hot (B5—colloidal solution; B6–B8 (scale bar: 200, 100, 10 nm)).

**FIGURE 12:**
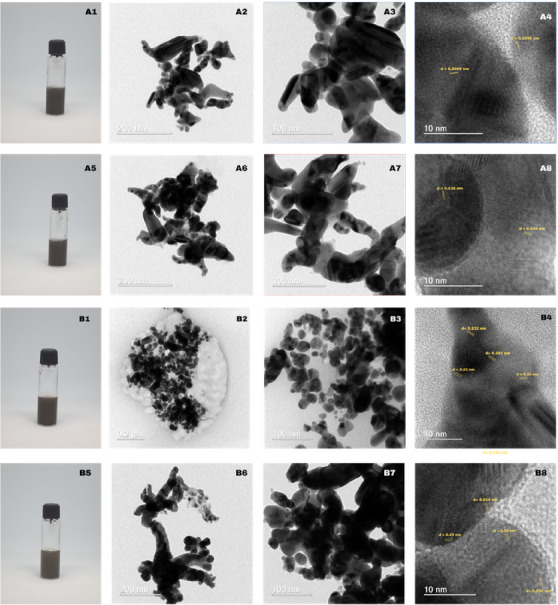
Colloidal solution image and HRTEM images for USE‐AgNP‐rt (A1—colloidal solution; A2–A4 (scale bar: 200, 100, 10 nm)) and USE‐AgNP‐hot (A5—colloidal solution; A6–A8 (scale bar: 200, 100, 10 nm)). GSE‐AgNP‐rt (B1—colloidal solution; B2–B4 (scale bar: 200, 100, 10 nm)) and GSE‐AgNP‐hot (B5—colloidal solution; B6–B8 (scale bar: 200, 100, 10 nm)).

Images obtained by HRTEM and photography of the colloidal solution of all prepared gold and silver nanomaterials are presented in Figures 11 and 12, respectively.

Figure 11 A1–A5 illustrates a systematic color transition from light pink‐rose (A1) to deeper magenta (A5) and dark violet‐purple (B1); B5 is slightly lighter than B1 yet darker than A1. Optical clarity correlates with color intensity: A1 and A5 remain relatively translucent, B1 shows the highest optical density, and B5 is intermediate [14]. Low‐magnification HRTEM in Figure 11 A2, A6 reveals percolating chains of near‐spherical Au nanoparticles (8–25 nm, log‐normal mean 14 nm) that coalesce into dense dendritic networks [12], whereas the B series in Figure 12 B2,B6 retains more isolated particles along branched chains, forming loose aggregates with minimal neck growth [15].

Intermediate‐magnification images in Figure 11 A3,A7, B3, B7 confirm quasi‐spherical to polyhedral shapes with high circularity (C 0.85) and no radially symmetric protrusions, indicating isotropic, surface‐energy‐driven growth. Sharp boundaries between adjoining particles show that aggregation occurs after crystallization rather than by continuous oriented attachment [16].

HRTEM shown in Figure 11 A4,A8,B4,B8 resolves lattice fringes with *d* 0.23 and *d* 0.20 nm, matching the {1 1 1} and {2 0 0} planes of fcc gold. Fringes span entire particles, confirming single‐crystal domains. In sample A, coherent fringes occasionally bridge short necks, evidencing partial oriented attachment and low‐angle boundary formation, whereas in sample B, fringes terminate at particle surfaces, consistent with simple physical aggregation without sintering [15].

No spacing attributable to Au oxides or alloyed phases is detected. Rare Σ3 twin planes (*<*5%) suggest that multiply twinned motifs are minor [16], and the dominant morphology is single‐crystalline, isotropic nanospheres.

Collectively, Figure 11 shows that both syntheses yield phase‐pure fcc Au nanocrystals with distinct assemblies: sample A forms densely fused dendritic networks, whereas sample B preserves discrete particles in open fractal clusters, influencing surface accessibility and interparticle plasmonic coupling.

In Figure 12, the progressive deepening from medium to dark grey correlates with the growth‐driven red‐shift of the Ag surface plasmon and thus with increasing optical density, a trend previously observed for room‐temperature dendritic growth of silver nanostructures [17].

The ramified, chain‐like aggregates of 20–80 nm single‐crystal subunits in sample A mirror the oriented‐attachment dendrites reported by Wen et al. [17], where primary particles fuse preferentially along low‐energy {1 1 1}/{1 0 0} facets to produce open three‐dimensional networks. Similar branch–stem interfaces attributed to facet‐selective attachment were analyzed in detail by Tang et al. [18], supporting the crystallographic continuity observed across necks in Figure 12A.

In contrast, the quasi‐spherical 15–40 nm crystallites decorating an amorphous matrix in sample B resemble the densely packed nanospheres obtained under kinetic control by Tsai et al. [19] and the plant‐reduced AgNPs of Suman et al. [20] that was characterized by limited oriented attachment. The narrower size dispersion of our B series further agrees with the ion‐irradiation‐refined Ag nanospheres described by Sherpa et al. [21].

HRTEM lattice spacings of 0.225, 0.232–0.243, and 0.200 nm match the fcc Ag {1 1 1}/{2 0 0} reflections in every sample, confirming phase purity across morphologies. Σ3 twin boundaries, hallmarks of multiply twinned decahedral or icosahedral motifs, are consistent with the structural models proposed by Wen et al. [17] for dendritic silver. Collectively, these comparisons place sample A within the oriented‐attachment dendrite regime and sample B within the quasi‐spherical, independently nucleated nanoparticle regime described in the literature [18].

## 4 Conclusion

Preprocessing pink‐pepper biomass by grinding and sonication, followed by mild heating, produces ultrasound‐assisted gold and silver nanoparticles with markedly superior colloidal stability over those obtained from untreated material. Particles derived from processed extracts are smaller, more uniform, more negatively charged, and less prone to aggregation. TEM reveals that these gold nanoparticles fuse into coherent dendritic frameworks through oriented attachment, whereas unprocessed gold and silver counterparts persist as discrete nanospheres held together only by weak adsorption. All particles are phase‐pure fcc, and FTIR shows strong phenolic hydroxyl and carbonyl coordination particularly in silver underpinning their enhanced stability. Mechanical milling thus offers a low‐toxicity lever for tuning nucleation, assembly, and physicochemical properties, enabling sustainable production of noble‐metal nanomaterials tailored for biomedical, catalytic, and environmental applications; further refinement of extraction protocols and mechanistic insight will elevate their performance even more.

## Conflicts of Interest

The authors declare no conflicts of interest.

## Data Availability

The data that support the findings of this study are available from the corresponding author upon reasonable request.
